# The influence of body composition on the response to dynamic stimulation of the endocrine pituitary-testis axis

**DOI:** 10.1038/s41366-024-01518-2

**Published:** 2024-04-12

**Authors:** Julie Abildgaard, Anne Kirstine Bang, Loa Nordkap, Lærke Priskorn, Niels Jørgensen

**Affiliations:** 1 Department of Growth and Reproduction, Copenhagen University Hospital, Rigshospitalet, Copenhagen, Denmark; 2 International Center for Research and Research Training in Endocrine Disruption of Male Reproduction and Child Health (EDMaRC), Copenhagen University Hospital, Rigshospitalet, Copenhagen, Denmark; 3 The Centre for Physical Activity Research, Rigshospitalet, University of Copenhagen, Copenhagen, Denmark

**Keywords:** Obesity, Endocrinology

## Abstract

**Background:**

Testosterone treatment is generally not recommended in men with obesity induced low serum testosterone. However, distinguishing this condition from overt testosterone deficiency in men with obesity where treatment should be initiated is a diagnostic challenge and tools to differentiate these conditions are scarce but could be of important clinical relevance.

**Objectives:**

To investigate the association between body composition and dynamic responses of the pituitary-testis axis in men.

**Methods:**

Single-center cross-sectional study including 112 healthy men. Participants went through a full biochemical assessment of the pituitary-testis axis, and dynamic stimulatory tests of luteinizing hormone (LH) secretion (gonadotropin-releasing hormone (GnRH)-test) and testosterone secretion (choriogonadotropin (hCG)-test). A subset (*N* = 78) further had a DXA-scan performed.

**Results:**

A higher body mass index (BMI) was associated with lower basal serum LH (B_U_ = −0.44, 95% CI: −0.88–−0.01, *p* = 0.04). The GnRH-stimulated LH increase was not significantly associated with BMI (B_U_ = −0.10, 95% CI: −0.72–0.51, *p* = 0.74). Furthermore, a high BMI was associated with low basal testosterone (B_U_ −0.02, 95% CI: −0.03–−0.02, *p* < 0.001), and free testosterone (B_U_ −15.0, 95% CI: −19.9–−10.0, *p* < 0.001) and men with overweight and obesity had significantly lower testosterone (9%, *p* = 0.003 and 24%, *p* < 0.001) and free testosterone (25%, *p* = 0.006 and 50%, *p* < 0.001) concentrations compared to men with normal weight. The HCG-stimulated testosterone increase was significantly less dependent on BMI compared to the influence of BMI on basal testosterone concentrations (*p* = 0.04 for the interaction).

**Conclusions:**

Dynamic sex hormone responses following pituitary-testis axis stimulation were less dependent on BMI, compared to the influence of BMI on basal hormone concentrations and could potentially assist clinical decision making in patients with obesity suspected of testosterone deficiency.

## Introduction

The prevalence of obesity is drastically increasing, reaching pandemic heights with more than 1 billion people worldwide living with obesity (https://www.who.int/news/item/04-03-2022-world-obesity-day-2022-accelerating-action-to-stop-obesity). Several studies suggest a strong association between serum testosterone (T) concentrations and excess body weight in men [1–3]. However, the interplay between body composition and functioning of the hypothalamic-pituitary-gonadal (HPG) axis is complex and not fully understood and several mechanisms are thought to contribute to obesity-induced inhibition of the HPG axis. Thus, obesity, particularly central obesity and steatosis, inhibits production of sex hormone binding globulin (SHBG) [4, 5] contributing to lower circulating total T, through a lower T binding capacity, with only little affection of the free testosterone (FT) concentration [5]. Furthermore, increased adipose tissue-related aromatization of T to estradiol (E_2_) is believed to contribute to lower serum T and higher E_2_, to a varying extent, potentially increasing negative feedback at the hypothalamo-pituitary level, whereby T production is inhibited [6, 7]. Lastly, obesity associated chronic inflammation and adipokine signaling has been suggested to impair gonadotropin secretion, Leydig cell function and T production [8].

T treatment is generally not recommended in cases of obesity-induced low serum T [9]. Thus, distinguishing this condition from overt T deficiency, where treatment should be initiated, is of clinical importance. However, this task is complicated and tools to assist clinical decision making are requested.

Dynamic testing of the HPG axis can be used to support the diagnostic process of T deficiency [10, 11]. The maximum gonadotropin production from the pituitary gland can be assessed by the gonadotropin-releasing hormone (GnRH) test, whereas the maximum capacity of the Leydig cells’ T production can be assessed by the human chorionic gonadotropin (hCG) test. However, it is largely unknown how body weight affects the HPG response upon stimulation. Thus, this study was conducted to investigate the association between body mass index (BMI) and dynamic responses of the HPG axis upon stimulation in a healthy cohort of men.

## Methods

### Study population

The study population was healthy men from the general Danish population examined between year 2012 and 2014. All men were participating in an ongoing study of testicular function, where the men underwent a general health examination, completed questionnaires concerning general health and lifestyle factors and a subset delivered a semen sample [11, 12]. The included men were in addition to standard testing, invited to take part in dynamic tests of the HPG axis. Participants who either had a hCG test, a GnRH test, or both performed within the test period were included in this study. A subset of the participants also went through a full-body DXA-scan. Exclusion criteria were 1) chronic diseases, 2) medical history of testicular surgery or trauma, 3) current use of anabolic steroids. All examinations, tests and laboratory assessments were performed at Department of Growth and Reproduction, Rigshospitalet, Copenhagen, Denmark. The study was approved by the ‘Ethical committee of the Capital region’ (permit number H-KF-289428) and performed according to the Declaration of Helsinki. Individual consent was obtained verbally and in writing from all participants.

### Reproductive hormone analyses

Serum concentrations of LH and T were determined using a time-resolved fluoroimmunoassay (Delfia, Wallac, Turku, Finland). E_2_ concentrations were determined using radioimmunoassay (Biotech-IGG, Pantex). SHBG was determined by time-resolved chemiluminescent immunoassay (Access, Beckman Coulter). Inter- and intraassay coefficients of variation (CVs) for measurements of the hormones for LH were 2 and 3%, SHBG 5 and 4%, T 10 and 6%, and for E_2_ 15 and 8%. FT was calculated using the Vermeulen formula with a fixed albumin (43.8 g/L) [13]. Total testosterone was considered as low with serum concentrations below 10 nmol/L and free testosterone was considered low at serum concentrations below 200 pmol/L based on laboratory specific reference ranges. All analyses were validated and accredited by the Danish Accreditation Fund (DANAK, www.danak.dk).

### HPG stimulation tests

Reproductive hormone testing was performed as described previously [11]. GnRH and hCG tests were initiated between 08:00 and 12:00 h. Initially, a common baseline blood sample for both the GnRH and hCG test was drawn for measurement of LH and T. LH and T increases were calculated as the difference between basal and stimulated serum concentrations as we previously showed that these measurements are a valuable diagnostic tool in the evaluation of HPG disorders [11]. *GnRH stimulation test:* 100 μg GnRH (Relefact, Sanofi-Aventis, Frankfurt, Germany) was given intravenously, and a blood sample was collected after 30 min for LH measurement. *HCG stimulation test:* Was initiated immediately following the GnRH test. An injection of 5000 IU hCG (Pregnyl, Organon, Amsterdam, Netherlands) was given in the gluteal muscle. A follow-up blood sample was taken 72 h later, with an allowed variation of +/−1 h, for measurement of T.

### Body composition

Fat and lean body masses were assessed through a DXA scan (Lunar Prodigy Advance; GE Medical Systems Lunar, Milwaukee, WI, USA). Software (Prodigy, enCORE 2004, version 8.8; GE Lunar Corp., Madison, WI, USA) was used to estimate regional and total fat and fat-free tissue masses. CVs were 2% for total body fat and regional body fat, measured on humans.

### Statistical analyses

Continuous variables are displayed as medians with interquartile ranges (IQR) unless otherwise stated. Data were log_10_-transformed when not normally distributed. Histograms and Q–Q plots were used to assess normal distribution. Analyses of the association between body composition and basal and stimulated sex hormone concentrations were performed using a linear regression model. Models were checked for assumptions of the linear model, including normal distribution of the residuals, linearity, and homogeneity of variance. If a statistically significant association was observed, a best-fit curve was added to the figure including 95% confidence intervals. Interaction analyses were performed to investigate whether the association between BMI and basal serum hormone concentrations differed significantly from that of BMI and stimulated serum hormone concentrations. Participants were subdivided into three subgroups based on BMI: 1) men with normal weight (BMI < 25 kg/m^2^), 2) overweight (BMI 25–29.9 kg/m^2^), and 3) obesity (BMI ≥ 30 kg/m^2^). Basal and stimulated serum sex hormone concentrations were compared between subgroups using a one-way ANOVA. A Bonferroni post-hoc test was performed to assess between group differences. A two-way ANOVA was performed to estimate the effects of two independent categorical variables (high/low BMI: BMI < 25 kg/m^2^ versus BMI ≥ 25 kg/m^2^ and high/low FT: FT < 200 pmol/L versus FT ≥ 200 pmol/L) on dynamic test responses of the HPG axis. Single mediation analyses were conducted using the PROCES plug-in (v4.2). Statistical analyses were performed using IBM SPSS statistics version 28. A *p*-value < 0.05 was considered statistically significant.

## Results

### Subject characteristics

Study participants had a median age of 30.5 years (IQR: 19.0–35.5) and a BMI of 24.0 kg/m^2^ (range: 17.3–59.1 kg/m^2^). A BMI < 25 kg/m^2^ was registered in 65 men, 29 had a BMI between 25–29.9 kg/m^2^, and 18 had a BMI ≥ 30 kg/m^2^. Median T was 16.4 nmol/L (IQR: 11.9–21.3 nmol/L), FT was 369 pmol/L (IQR: 256–493 pmol/L) and LH 3.4 IU/L (IQR: 2.6–4.5 IU/L). Twenty-one men had a serum T considered low (T < 10 nmol/L) and 19 men had a FT considered low (FT < 200 pmol/L). All men were Danish citizens. Subject characteristics are shown in Table 1.

**Table 1:** Subject characteristics.

Total (*N*)	*N*	112
Age, years	112	30.5 (19.0–35.5)
S-T, nmol/L	112	16.4 (11.9–21.3)
S-E_2_, pmol/L	92	70 (51–89)
S-SHBG, nmol/L	111	28.6 (22.6–36.1)
S-FT, pmol/L	112	369 (256–493)
S-LH, IU/L	112	3.4 (2.6–4.5)
BMI, kg/m^2^	112	24.0 (22.0–26.2)
BMI, group _(<25/25.0-29.9/≥30.0)_, N	112	65/29/18
Lean body mass, kg	78	61.9 (56.3–65.4)
Fat mass, kg	78	15.9 (12.8–21.9)
Trunk fat, kg	78	7.7 (5.5–11.7)
Limb fat, kg	78	7.4 (6.1–9.2)

Subject characteristics of study participants presented as median (interquartile range).

*S* serum, *T* testosterone, *E*_*2*_ estradiol, *SHBG* sex hormone binding globulin, *FT* free testosterone, *BMI* body mass index, *LH* Luteinizing hormone.

### Association between BMI, and basal LH and GnRH-stimulated LH increase

**Fig. 1:**
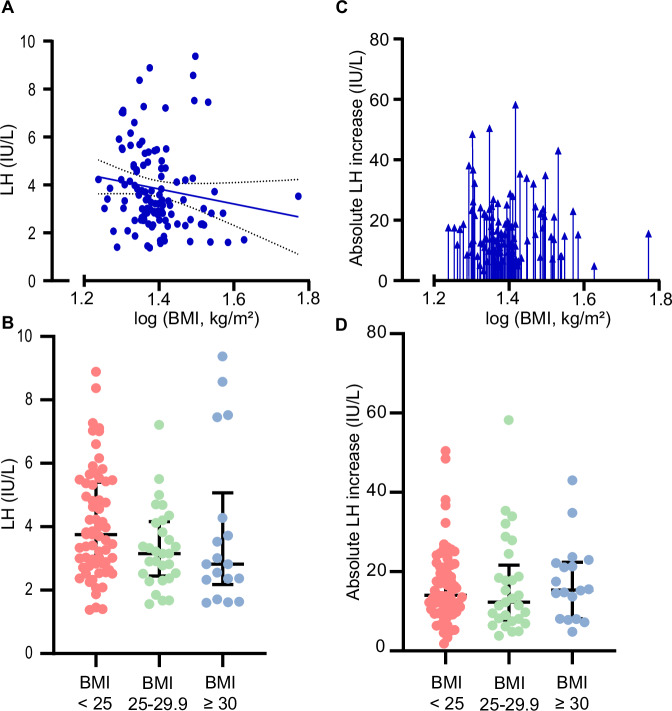
Basal serum luteinizing hormone (LH) and gonadotropin-releasing hormone (GnRH)-stimulated LH increase in relation to body mass index (BMI) in men. **A** Basal LH in relation to BMI in healthy men (*n* = 112). **B** Basal LH in men with normal weight (BMI < 25 kg/m^2^, *n* = 65), overweight (BMI 25–29.9 kg/m^2^, *n* = 29), and obesity ( ≥ 30 kg/m^2^, *n* = 18). **C** The GnRH-stimulated LH increase in relation to BMI in healthy men (*n* = 112). **D** The GnRH-stimulated LH increase in men with normal weight (BMI < 25 kg/m^2^, *n* = 65), overweight (BMI 25–29.9 kg/m^2^, *n* = 29), and obesity ( ≥ 30 kg/m^2^, *n* = 18). Every dot or arrow represents one person. Line represents a significant association including the 95% confidence interval.

BMI explained 4% of the variation in basal LH (B_U_ = −0.44, 95% CI: −0.88–−0.01, *p* = 0.04), where a higher BMI was associated with lower basal LH (Fig. 1A). A single-mediation analysis revealed that 14% (95% CI: 1–36%) of the negative relationship between BMI and LH was statistically mediated through serum E2. Basal LH concentrations did not differ significantly between BMI sub-groups (*p* = 0.16) (Fig. 1B). BMI was not significantly associated with the GnRH-stimulated LH increase (B_U_ = −0.10, 95% CI: −0.72–0.51, *p* = 0.74) (Fig. 1C) and GnRH-stimulated LH concentrations did not differ between BMI sub-groups (*p* = 0.70) (Fig. 1D).

### Association between BMI, and basal T, and HCG-stimulated T increase

**Fig. 2:**
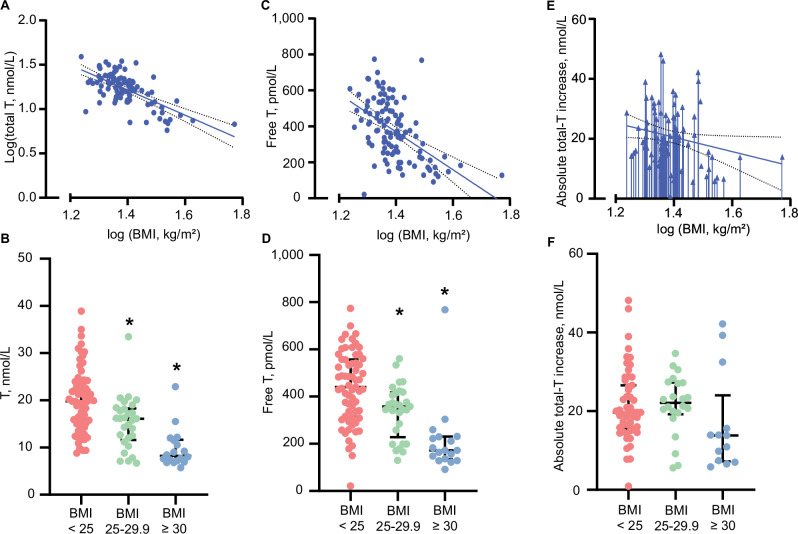
Basal serum testosterone (T), free testosterone (FT) and choriogonadotropin (HCG)-stimulated T increase in relation to body mass index (BMI) in men. **A** Basal T in relation to BMI in healthy men (*n* = 112). **B** Basal T in men with normal weight (BMI < 25 kg/m^2^, *n* = 65), overweight (BMI 25–29.9 kg/m^2^, *n* = 29), and obesity (≥30 kg/m^2^, *n* = 18). **C** Basal FT in relation to BMI in healthy men (*n* = 112). **D** Basal FT in men with normal weight (BMI < 25 kg/m^2^, *n* = 65), overweight (BMI 25–29.9 kg/m^2^, *n* = 29), and obesity (≥30 kg/m^2^, *n* = 18). **E** The HCG-stimulated T increase in relation to BMI in healthy men (*n* = 91). **F** The HCG-stimulated T increase in men with normal weight (BMI < 25 kg/m^2^, *n* = 55), overweight (BMI 25–29.9 kg/m^2^, *n* = 22), and obesity (≥30 kg/m^2^, *n* = 14). Every dot or arrow represents one person. Line represents a significant association including the 95% confidence interval. T testosterone. *Significantly different from normal weight, *p* < 0.05.

BMI explained 35% of the variation in basal serum T (B_U_ −0.02, 95% CI: −0.03–−0.02, *p* < 0.001), where a higher BMI was associated with a lower basal serum T (Fig. 2A). Serum T was 9% (95% CI: 5–14%, *p* = 0.003) lower in men with overweight and 24% (95% CI: 19–30%, *p* < 0.001) lower in men with obesity compared to men with normal weight (Fig. 2B). Single-mediation analyses revealed that 17% (95% CI: 5–33%) of the negative relationship between BMI and testosterone was statistically mediated through serum SHBG. BMI explained 25% of the variation in FT (B_U_ −15.0, 95% CI: −19.9–−10.0, *p* < 0.001), where a higher BMI was associated with a lower basal serum FT (Fig. 2C). Serum FT was 25% (95% CI: 16–34%, *p* = 0.006) lower in men with overweight and 50% (95% CI: 39–62%, *p* < 0.001) lower in men with obesity compared to men with normal weight (Fig. 2D). BMI explained 5% of the variation in the HCG-stimulated T increase (B_U_ −0.36, 95% CI: −0.69–−0.03, *p* = 0.04, Fig. 2E). The relationship between BMI and HCG-stimulated testosterone was not statistically mediated through SHBG (20%, 95% CI: −12–71%). The negative association between BMI and basal T was significantly steeper compared to the negative association between BMI and HCG-stimulated T (*p* = 0.04 for the interaction). HCG-stimulated T was not significantly different in men with overweight (0%, 95% CI: −15–17%, *p* = 0.99) and obesity (−21%, 95% CI: −57–14%, *p* = 0.26) compared with men with normal weight (Fig. 2F).

### Dynamic sex hormone responses in men with low versus normal FT

**Fig. 3:**
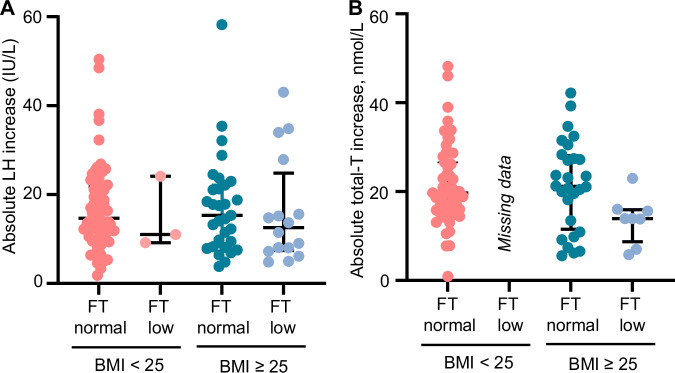
Gonadotropin-releasing hormone (GnRH)-stimulated LH increase and choriogonadotropin (HCG)-stimulated testosterone (T) increase in men stratified by body mass index (BMI) and free testosterone (FT). **A** The GnRH-stimulated LH increase in men with normal FT, BMI < 25 kg/m^2^: *n* = 62. Low FT, BMI < 25 kg/m^2^: *n* = 3. Normal FT, BMI ≥ 25 kg/m^2^: *n* = 31. Low FT, BMI ≥ 25 kg/m^2^: *n* = 16. **B** The HCG-stimulated T increase in men with normal FT, BMI < 25 kg/m^2^: *n* = 55. Low FT, BMI < 25 kg/m^2^: *n* = 0 (missing data). Normal FT, BMI ≥ 25 kg/m^2^: *n* = 28. Low FT, BMI ≥ 25 kg/m^2^: *n* = 8.

The GnRH-stimulated LH increase in men with low basal FT did not differ significantly from men with normal basal FT (*p* = 0.41 for effect of FT), in either men with a normal BMI or men with a higher BMI (*p* = 0.96 for interaction) (Fig. 3A). Accordingly, the HCG-stimulated T increase in men with low basal FT did not differ significantly from men with normal basal FT (*p* = 0.21 for effect of FT). Interaction analyses could not be performed due to lack of data on men with normal weight and low FT (Fig. 3B).

### Association between fat distribution, and basal and stimulated LH and T

We used DXA-scans to investigate the association between regional adiposity and basal versus stimulated sex hormone responses. Trunk fat was significantly associated with basal LH (B_U_ = −0.02, 95% CI: −0.03–0.00, *p* = 0.03), total T (B_U_ = −0.02, 95% CI: −0.03–0.00, *p* = 0.02), and FT (B_U_ = −15.0, 95% CI: −27.2– −2.7, *p* = 0.02), where a higher trunk fat mass was associated with lower basal serum LH, T, and FT. Trunk fat mass was not significantly associated with GnRH-stimulated LH (B_U_ = −0.77, 95% CI: −1.69–0.14, *p* = 0.10) or HCG-stimulated T (B_U_ = −0.45, 95% CI: −1.33–0.43, *p* = 0.31). Limb fat was not significantly associated with either basal or stimulated LH or T (Table 2).

**Table 2:** Associations between regional adiposity and basal and stimulated serum LH and T.

	Basal LH, IU/L		GnRH-stimulated LH, IU/L		Basal T, nmol/L		Basal FT, pmol/L		HCG-stimulated T increase, nmol/L	
	B (95% CI)	*p*-value	B (95% CI)	*p*-value	B (95% CI)	*p*-value	B (95% CI)	*p*-value	B (95% CI)	*p*-value
Trunk fat, kg	−0.02 (−0.03–−0.00)	0.03	−0.77 (−1.69–0.14)	0.10	−0.02 (−0.03–0.00)	0.02	−17.0 (−30.5–−3.4)	0.02	−0.45 (−1.33–0.43)	0.31
Limb fat, kg	0.02 (−0.01–0.04)	0.18	1.11 (−0.42–2.64)	0.15	0.01 (−0.01–0.03)	0.37	13.0 (−9.7–35.7)	0.26	1.40 (−0.21–3.00)	0.09

Multiple regression analysis: Rows: Independent variables. Columns: dependent variables.

*LH* luteinizing hormone, *GnRH* Gonadotropin-releasing hormone, *T* testosterone, *FT* free testosterone, *HCG* human choriogonadotropin.

## Discussion

Distinguishing obesity-induced low concentrations of total T from overt T deficiency, where T treatment is indicated, can be a challenging task. Diagnostic tools to assist clinical decisions are therefore highly requested. We previously showed that dynamic testing of the HPG axis can be used as a tool to support the diagnostic process of T deficiency [11]. However, the effect of body composition on these tests have remained unknown.

In this study of 112 healthy men, we found that the dynamic sex hormone responses following HPG axis stimulation were less dependent on BMI, compared to the influence of BMI on basal serum sex hormone concentrations. Thus, a high BMI was associated with low basal LH, but the GnRH-stimulated LH increase was not significantly associated with body composition. Furthermore, the negative association between BMI and basal serum T was significantly steeper compared to the negative association between BMI and the HCG-stimulated T increase.

In accordance with previous studies, we detected that both basal total T and FT was closely associated with BMI [14–17]. Stratifying basal T concentrations by BMI class confirmed a stepwise decrease in basal T and FT with increasing BMI class, whereas the HCG-stimulated T increase was not significantly different in men with either overweight or obesity compared to men with normal weight. However, we did see a trend towards a lower HCG-stimulated T increase in a subgroup of the men with obesity, suggesting a considerable individual variation in the effects of adipose inhibition on the dynamic response of the pituitary-testis axis, within this group. Several studies indicate that the metabolic burden related to obesity varies substantially among individuals [18–20]. Thus, up to 25–50% of people with obesity are estimated to have a metabolically healthy phenotype, despite the higher body weight [21]. We speculate, that obesity with a higher metabolic burden (metabolically unhealthy obesity) could impact dynamic pituitary-gonadal responses to a greater extent, as a high trunk fat mass was associated with lower serum sex hormone concentrations whereas limb fat was not. This is in accordance with previous studies indicating a particularly significant role of visceral fat in the adipose inhibition of the HPG axis, beyond steroid hormone aromatization and regulation of SHBG [22–25]. We found, that serum E_2_ and SHBG only partly statistically mediated basal sex hormone concentrations and did not statistically mediate dynamic sex hormone responses, suggesting that additional mechanisms contribute to the crosstalk between adipose tissue and the HPG axis. In relation to this, obesity-related inflammation has been shown to inhibit HPG-signaling and blockade of central inflammatory pathways, in men with obesity and low serum T, increase T concentrations [26]. Several studies further suggest substantial adipokine signaling from the adipose tissue to the HPG axis through e.g., leptin and adiponectin [27–29].

The association between BMI and LH was generally modest compared to the association between BMI and testosterone. Previous studies suggest that severe obesity (BMI ≥ 40 kg/m^2^) is required to suppress pituitary gonadotropin secretion [30, 31]. In this study, only two men had a BMI ≥ 40 kg/m^2^ which prevent us from drawing conclusions in cases of class III obesity. However, one previous study of 10 men with class III obesity confirms substantial stimulatory HPG axis reserve in this group of men as well [32]. Nonetheless, we did find a significant association between a higher BMI and lower basal LH which was partly statistically mediated through higher serum E_2_. While studies indicate that serum E_2_ concentrations directly reflect the negative feedback exerted by estrogens on gonadotropin release, the effects of excess adiposity on serum E_2_ remains debated [33–35].

Obesity is associated with several endocrine abnormalities arising from changes in the hypothalamo-pituitary axis [36]. For example, obesity attenuates the stimulated growth hormone response [37], leads to ACTH hyperresponsiveness [38], and is associated with thyroid dysfunction [39]. Thus, it is no surprise that also the interpretation of the HPG axis functioning is complicated by obesity.

Whether T treatment is beneficial in men with obesity and low serum T concentrations is controversial [9, 40, 41]. Considering the limited number of studies showing beneficial effects, T therapy is currently not recommended in the prevention of metabolic disturbances associated with obesity and guidelines recommend that only patients with low FT and symptoms of T deficiency should be considered T deficient [3, 9].

This study was performed on healthy men with no symptoms of T deficiency. In a clinical setting, a patient might have T deficiency, which could impact the relationship between body composition and HPG axis functioning differently [42]. However, the most common challenge is to identify and sort out the man with obesity-induced low T, who does not need further T treatment. The findings of this study indicate that dynamic testing of the HPG axis could be a valuable tool to identify the otherwise healthy man with obesity-related low serum T, because stimulation of the HPG axis seems less sensitive to body composition. Thus, a normal response of the GnRH-test as well as the HCG-test in a man with overweight or obesity will suggest that his low serum T concentration is due to excess adiposity rather than overt T deficiency. Thus, our results are, to the best of our knowledge, the first to evaluate the potential impact of overweight and obesity on the dynamic sex hormone stimulation tests and the first to provide suggestions for a tool to differentiate between obesity-induced low serum T concentrations versus overt T deficiency in men with excess bodyweight.

Our study has some limitations. The study was based on observational data and, consequently, causal conclusions could not be drawn. Only 18 out of 112 men had a BMI ≥ 30 kg/m^2^, which affects the precision of the estimates in men with obesity. Furthermore, low FT in men with normal weight was rare and conclusions on dynamic test responses in this group could therefore not be drawn. Sex hormone measurements were performed using immunoassays. Whereas this only impacts accuracy of serum T measurements, within the normal range, to a small extent, serum concentrations of E_2_ measured using immunoassays are less accurate compared to mass spectrometry [43]. Furthermore, FT was calculated from Vermeulen’s formula, taking serum SHBG and albumin into account, and not measured from equilibrium dialysis, the gold standard. However, these circumstances often reflect the clinical reality, where advanced mass spectrometry and equilibrium dialysis equipment is not available. Excessive fat mass might inhibit hypothalamic secretion of GnRH through negative feedback from E_2_ [44]. We did not have data from clomiphene testing and therefore could not investigate the effects of bodyweight on dynamic sex hormone secretion at the hypothalamic level.

In conclusion, basal sex hormone concentrations are closely associated with BMI making the diagnostic evaluation of T deficiency complicated in patients with concomitant overweight and obesity. Dynamic sex hormone responses following HPG axis stimulation are less dependent on BMI and may therefore serve as a potentially valuable tool to assist clinical decision making in patients with overweight and obesity suspected of T deficiency.

## Data Availability

The datasets analyzed during the current study are not publicly available due to national data security legislation but are available from the corresponding author on reasonable request and with permission from the ethical committee of the capital region of Denmark.
