# Correction: The influence of body composition on the response to dynamic stimulation of the endocrine pituitary-testis axis

**DOI:** 10.1038/s41366-024-01583-7

**Published:** 2024-07-31

**Authors:** Julie Abildgaard, Anne Kirstine Bang, Loa Nordkap, Lærke Priskorn, Niels Jørgensen

**Affiliations:** 1 Department of Growth and Reproduction, Copenhagen University Hospital, Rigshospitalet, Copenhagen, Denmark; 2 International Center for Research and Research Training in Endocrine Disruption of Male Reproduction and Child Health (EDMaRC), Copenhagen University Hospital, Rigshospitalet, Copenhagen, Denmark; 3 The Centre for Physical Activity Research, Rigshospitalet, University of Copenhagen, Copenhagen, Denmark

**Keywords:** Obesity, Endocrinology

Correction to: *International Journal of Obesity* 10.1038/s41366-024-01518-2, published online 12 April 2024

An incorrect version of Figure 3, based on erroneous testosterone cut-offs, was mistakenly published.

Figure 3 in the original article:
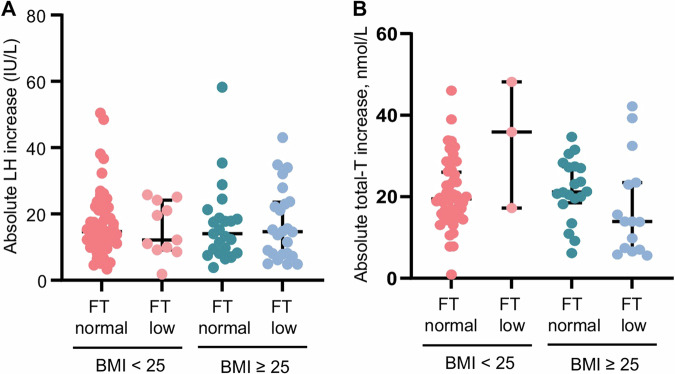


Corrected Figure 3:
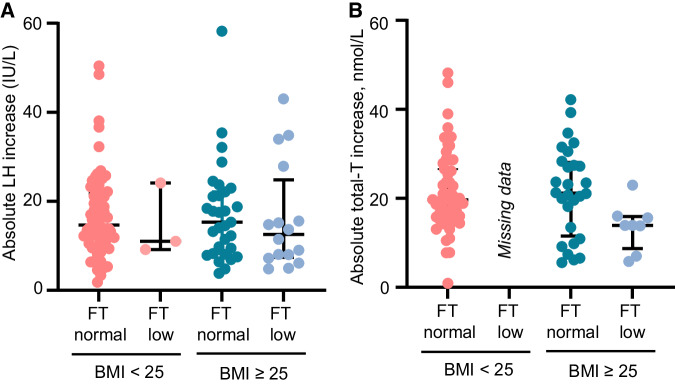


The original article has been corrected.

